# Isotopic Investigation of Contemporary and Historic Changes in Penguin Trophic Niches and Carrying Capacity of the Southern Indian Ocean

**DOI:** 10.1371/journal.pone.0016484

**Published:** 2011-02-02

**Authors:** Audrey Jaeger, Yves Cherel

**Affiliations:** Centre d’Etudes Biologiques de Chizé, Centre National de la Recherche Scientifique, Villiers-en-Bois, France; National Institute of Water & Atmospheric Research, New Zealand

## Abstract

A temperature-defined regime shift occurred in the 1970s in the southern Indian Ocean, with simultaneous severe decreases in many predator populations. We tested a possible biological link between the regime shift and predator declines by measuring historic and contemporary feather isotopic signatures of seven penguin species with contrasted foraging strategies and inhabiting a large latitudinal range. We first showed that contemporary penguin isotopic variations and chlorophyll a concentration were positively correlated, suggesting the usefulness of predator δ^13^C values to track temporal changes in the ecosystem carrying capacity and its associated coupling to consumers. Having controlled for the Suess effect and for increase CO_2_ in seawater, δ^13^C values of Antarctic penguins and of king penguins did not change over time, while δ^13^C of other subantarctic and subtropical species were lower in the 1970s. The data therefore suggest a decrease in ecosystem carrying capacity of the southern Indian Ocean during the temperature regime-shift in subtropical and subantarctic waters but not in the vicinity of the Polar Front and in southward high-Antarctic waters. The resulting lower secondary productivity could be the main driving force explaining the decline of subtropical and subantarctic (but not Antarctic) penguins that occurred in the 1970s. Feather δ^15^N values did not show a consistent temporal trend among species, suggesting no major change in penguins’ diet. This study highlights the usefulness of developing long-term tissue sampling and data bases on isotopic signature of key marine organisms to track potential changes in their isotopic niches and in the carrying capacity of the environment.

## Introduction

The warming of Earth’s climate since the 1970s has been at a rate greater that at any other time in the last thousand years, producing sharp biological and ecological consequences [1]. Biodiversity is currently being lost at unprecedented rates due to human activities and climate change, with ∼25% of mammals and ∼12% of birds being globally threatened [2]. Sphenisciformes and Procellariiformes are among the world’s most endangered orders of birds [2], with many penguin, petrel and albatross populations declining in the Southern Ocean over the last decades [3], [4], [5]. Concurrent temporal variations of different southern seabird populations with contrasted foraging ecology (diving and flying predators) and living at different localities (from subtropical to Antarctic waters) suggest common environmental driving causes. These population variations coincided with an atmospheric temperature-defined regime shift in the 1970s in the southern Indian Ocean [4], [6], probably in response to a change in meridional atmospheric circulation [7]. The underlying causes of the population changes remain however unknown, because of the lack of long-term data sets on oceanic physical and biological parameters during that period [6], [8]. The links between demographic, ecological and environmental variables remain therefore elusive.

The usefulness of stable isotope analysis to reconstruct the ecological history of top predators has been highlighted when applied on a long-term series of biological samples [9], [10], [11], [12]. Since feathers can be safely collected on both live birds and museum specimens, feather isotopic signatures were used to depict long-term changes in birds’ feeding ecology [13], [14], [15]. The basic underlying principle is that the isotopic composition of feathers reflects feeding ecology during moult because keratin is metabolically inert after synthesis [16]. In the marine environment, the isotopic signature of seabird tissues is a powerful tool to investigate their trophic niche along two dimensions, with δ^13^C and δ^15^N values reflecting the consumers’ foraging habitat and trophic level, respectively [17]. δ^13^C values of predators were also used as a proxy for ecosystem primary productivity (carrying capacity, [18]), thus allowing investigating long-term ecosystemic changes using museum specimens [10], [15].

The main objective of the present study was to use the stable isotope method to depict possible changes in the feeding ecology of southern seabirds and/or in their foraging environment that could be linked to the 1970s atmospheric regime shift in the Southern Indian Ocean [4]. In agreement with an ecosystemic explanation, we expected a common isotopic pattern among species when compared samples collected before, during and after the regime shift. For doing this, we sampled archived museum specimens (historic records) together with living animals in the field (contemporary records). We focused on adult penguins for several practical and ecological reasons:

1. Unlike large Procellariiformes and penguin chicks, specimens of adult penguins are relatively numerous in museums [15].
2. Adult penguins moult immediately before or after breeding, during the productive austral summer, and not during the less productive austral winter as most flying birds do.
3. Moult is the most critical period of the penguin cycle, because energetic constraints preclude them moulting at sea [19]. Penguins renew their plumage while fasting on land. Consequently, their feather isotopic signature reflects the penguin isotopic niche during a well-defined temporal and spatial window, i.e. the restricted pre-moult feeding period during which they cannot disperse over wide marine areas.
4. Penguin species have contrasted foraging strategies and they live from Antarctica to the subtropics (e.g. [20], [21]). Concurrent sampling of different species living in different marine environments therefore make penguins ideal models to trace ecosystemic changes at various spatial scales.
5. Penguin moult involves two distinct processes, new feather synthesis and old feather loss that overlap in mid-moult [19]. We therefore collected both new and old feathers from the same individual penguins to test the assumption that the δ^13^C value of predators is a good proxy for ecosystem primary productivity (carrying capacity [18]). For doing this, we compared inter-annual differences in δ^13^C feather values to inter-annual changes in surface chlorophyll a (Chl a) concentrations (a proxy of primary productivity in the pelagic ecosystem, [22], [23]).

## Materials and Methods

### Ethics statement

Animals in this study were cared for in accordance with the guidelines of the ethics committee of the French Polar Institute (Institut Paul Emile Victor – IPEV) that approved all our fieldwork (program n° 109).

### Feather samples

**Figure 1:**
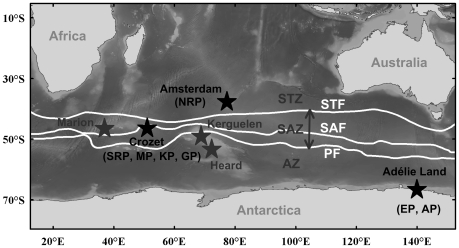
Map of the southern Indian Ocean with the main islands, oceanic fronts and zones. In black, sampling localities, with the sampled penguin species in parentheses. Abbreviations: NRP, northern rockhopper penguin; SRP, southern rockhopper penguin; MP, macaroni penguin; KP, king penguin; GP, gentoo penguin; EP, emperor penguin; AP, Adélie penguin; STF, Subtropical Front; SAF, Subantarctic Front; PF, Polar Front; STZ, Subtropical Zone; SAZ, Subantarctic Zone; AZ, Antarctic Zone [24].

Field study was conducted on the seven penguin species inhabiting the Terres Australes et Antarctiques Françaises: the emperor (EP, *Aptenodytes forsteri*) and Adélie (AP, *Pygoscelis adeliae*) penguins breeding in Adélie Land (Antarctica, 66°4’S, 140°0’E); the king (KP, *Aptenodytes patagonicus*), gentoo (GP, *Pygoscelis papua*), macaroni (MP, *Eudyptes chrysolophus*) and southern rockhopper (SRP, *Eudyptes chrysocome filholi*) penguins breeding at Possession Island (Crozet archipelago, 46° S, 52° E); and the northern rockhopper penguin (NRP, *Eudyptes moseleyi*) breeding at Amsterdam island (37°5’S, 77°3’E). Amsterdan Island is located north of the Subtropical Front (STF) in the Subtropical Zone (STZ), Crozet Islands lay between the STF and Polar Front (PF) within the Subantarctic Zone *sensus lato* (SAZ) and Adélie Land is positioned further south, in the high-Antarctic waters of the Antarctic Zone (AZ) (Fig. 1, [24]).

Contemporary feathers (old and new) were collected on live moulting adult penguins (Table 1). Old and new feathers refer to moults that occurred during the 2005–2006 and 2006–2007 austral summers (here called 2006 and 2007), respectively. Since EP moults in remote icy areas, this species was sampled during the 2007 breeding (not moulting) period. For both NRP and SRP, 30 additional contemporary samples collected in 2000 on live penguins were added to the data set for long-term analyses only (Tables 2 and 3). Historic feather samples (from 1875 to 1977) were obtained from specimens held in the ornithological collection of the Muséum National d’Histoire Naturelle of Paris (France). Feathers were taken only when complete capture information was present with a particular study specimen (N = 111). As colour (e.g. melanin) of feathers modifies slightly their stable isotopic ratios [25], only white breast body feathers were analysed in the present study.

**Table 1:** Feather isotopic signatures of seven penguin species in 2006 and 2007, and pair-wise isotopic differences between the two years.

			δ^13^C (‰)				δ^15^N (‰)		
	N per year	2006	2007	2007–2006	*p* value	2006	2007	2007–2006	*p* value
Emperor penguin	17	-	−23.1±0.3	-	-	-	12.1±0.3	-	-
Adélie penguin	10	−24.2±0.4	−23.4±0.4	0.8±0.5	0.001	9.4±0.4	10.7±0.6	1.3±0.5	<0.0001
King penguin	12	−22.0±0.5	−20.7±0.6	1.3±0.6	0.0001	11.4±0.3	11.3±0.5	−0.1±0.4	0.557
Gentoo penguin	12	−19.5±0.5	−19.1±0.2	0.4±0.2	0.002	10.4±1.0	11.3±0.9	0.9±0.4	0.0001
Macaroni penguin	12	−21.4±0.3	−21.1±0.3	0.3±0.3	0.006	9.8±0.3	9.7±0.3	−0.1±0.3	0.720
Southern rockhopper penguin	12	−21.2±0.3	−21.1±0.2	0.1±0.2	0.110	9.2±0.4	8.9±0.4	−0.3±0.4	0.099
Northern rockhopper penguin	12	−18.4±0.3	−17.9±0.2	0.5±0.3	0.0002	10.3±0.3	11.3±0.4	1.0±0.4	<0.0001

Values are mean ± SD. *P* values correspond to paired *t-*tests performed between the two years. Carbon isotopic values were not corrected for Suess and increase [CO_2_]_aq_ effects.

**Table 2:** Historic (1900s, 1950s, 1970s) and contemporary (2000s) carbon isotopic signatures of penguin feathers.

	1900s		1950s		1970s		2000s	
	N	δ^13^C (‰)	N	δ^13^C (‰)	N	δ^13^C (‰)	N	δ^13^C (‰)
Emperor penguin			11	−23.1±0.4 ^a^	3	−23.2±0.2 ^a^	17	−22.9±0.3 ^a^
				−23.2 (−23.7–−22.3)		−23.2 (−23.5–−23.1)		−22.9 (−23.4–−22.3)
Adélie penguin			5	−24.2±0.4 ^a^	3	−23.9±1.1 ^a^	20	−23.6 ±0.5 ^a^
				−24.4 (−24.5–−23.7)		−24.1 (−24.8–−22.6)		−23.6 (−24.6–−22.8)
King penguin					16	−20.5±0.6 ^a^	24	−20.7±0.9 ^a^
						−20.6 (−21.3–−19.5)		−20.6 (−22.0–−19.0)
Gentoo penguin					12	−19.2±1.2 ^a^	24	−18.5±0.4 ^b^
						−19.6 (−20.6–−17.6)		−18.4 (−19.6–−17.8)
Macaroni penguin					18	−20.8±0.6 ^a^	24	−20.5±0.4 ^b^
						−20.9 (−21.5–−20.0)		−20.5 (−21.0–−19.3)
Southern rockhopper penguin	2	−20.4 and −19.8			19	−20.9±0.5 ^a^	54	−20.5±0.4 ^b^
						−20.9 (−21.9–−20.3)		−20.5 (−21.0–−19.2)
Northern rockhopper penguin	8	−17.4±0.6 ^a^	2	−18.3 and −17.8	12	−18.9±1.0 ^b^	54	−17.6±0.5 ^a^
		−17.3 (−18.4–−16.5)				−19.1 (−20.1–−17.2)		−17.6 (−18.7–−16.5)

First line: values are mean ± SD; second line: median, minimum and maximum values. Values in the same line not sharing a common superscript letter are significantly different (*p*<0.05; see text). Carbon isotopic values were corrected for Suess and increase [CO_2_]_aq_ effects.

**Table 3:** Historic (1900s, 1950s, 1970s) and contemporary (2000s) nitrogen isotopic signatures of penguin feathers.

	1900s		1950s		1970s		2000s	
	N	δ^15^N (‰)	N	δ^15^N (‰)	N	δ^15^N (‰)	N	δ^15^N (‰)
Emperor penguin			11	12.6±0.5 ^a^	3	13.0±0.3 ^a^	17	12.1±0.3 ^b^
				12.6 (11.4–13.2)		13.0 (12.7–13.2)		12.0 (11.6–12.7)
Adélie penguin			5	10.1±1.6 ^a^	3	10.5±1.3 ^a^	20	10.1±0.8 ^a^
				9.3 (8.5–12.1)		10.3 (9.2–11.8)		9.9 (8.5–11.3)
King penguin					16	11.5±0.4 ^a^	24	11.3±0.4 ^a^
						11.6 (10.4–12.0)		11.3 (10.7–12.3)
Gentoo penguin					12	10.9±1.0 ^a^	24	10.8±1.0 ^a^
						11.0 (9.4–12.3)		10.9 (9.0–12.4)
Macaroni penguin					18	10.2±0.7 ^a^	24	9.8±0.3 ^b^
						10.2 (8.7–11.5)		9.8 (9.1–10.2)
Southern rockhopper penguin	2	10.9 and 10.7			19	9.2±0.6 ^a^	54	9.0±0.5 ^a^
						9.0 (8.4–10.4)		9.0 (7.8–10.0)
Northern rockhopper penguin	8	10.6±0.9 ^a^	2	11.2 and 10.3	12	11.1±0.6 ^a^	54	11.0±0.5 ^a^
		10.8 (8.7–11.7)				11.1 (10.2–12.2)		11.1 (9.8–12.0)

First line: values are mean ± SD; second line: median, minimum and maximum values. Values in the same line not sharing a common superscript letter are significantly different (*p*<0.05; see text).

### Stable isotope analysis

Prior to isotopic analysis, the tip (oldest part) of each feather was cut and discarded, because penguin feather synthesis begins at sea (review in [19]), and the use of dietary inputs vs. endogenous reserves influences feather δ^15^N values [26]. Two feathers of each individual penguin were cleaned of surface lipids and contaminants using a 2∶1 chloroform:methanol solution during 2 min following by two successive methanol rinses. They were then air dried and homogenised by cutting them into small fragments. Sub-samples were weighed (∼0.4 mg) with a microbalance, packed in tin containers, and nitrogen and carbon isotope ratios were subsequently determined by a continuous flow mass spectrometer (Micromass Isoprime) coupled to an elemental analyser (Euro Vector EA 3024). Stable isotope concentrations were expressed in conventional notation (δX  =  [R_sample_/R_standard_) −1] x 1000) where X is ^13^C or ^15^N and R is the corresponding ratio ^13^C/^12^C or ^15^N/^14^N. R_standard_ are PeeDee Belemnite and atmospheric N_2_ for δ^13^C and δ^15^N, respectively. Replicate measurements of internal laboratory standards (acetanilide) indicated measurement errors <0.15‰ and <0.20‰ for δ^13^C and δ^15^N, respectively.

### Data analysis

**Figure 2:**
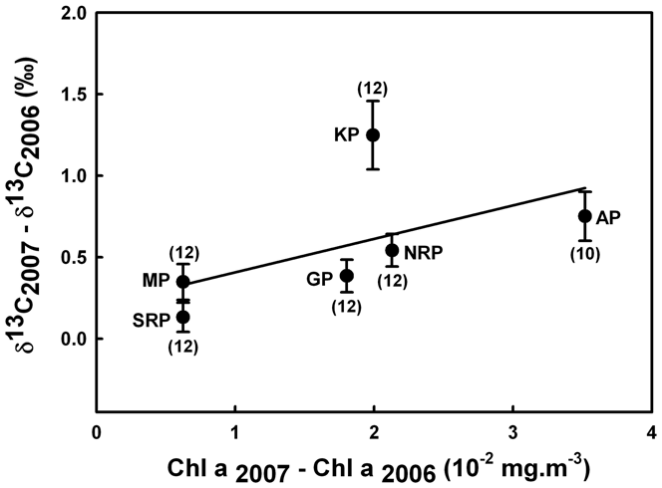
Correlation between inter-annual differences in penguin featherδ^13^C values and in sea-surface Chl a (corresponding years). Values are mean ± SE. Numbers in parentheses indicate sample sizes (N = 70). Solid line represents the linear regression (y = 20.87x+0.20, *r* = 0.354 and *p* = 0.003). Abbreviations: AP, Adélie penguin; GP, gentoo penguin; KP, king penguin; MP, macaroni penguin; NRP and SRP, northern and southern rockhopper penguins, respectively.

Differences in the δ^13^C and δ^15^N values of penguin feathers over the short term (2006 and 2007) were assessed using paired *t*-tests, because data followed Gaussian distributions (Table 1). A possible effect of ecosystem carrying capacity on penguin δ^13^C values was tested by looking at surface Chl a concentrations measured by satellite and provided by NASA (http://reason.gsfc.nasa.gov/Giovanni/) during both 2006 and 2007. For each year, Chl a concentration data were extracted and averaged from a square centred on the islands (10° longitude per 10° latitude) and for a period of two months before the initiation of the moulting fast on land (Table S1). The correlation between individual inter-annual differences in δ^13^C and Chl a was subsequently assessed using a Pearson’s correlation test (Fig. 2).

Historic samples were not regularly collected over time, with most of the penguins sampled in the 1950s and 1970s, a few in the 1900s and none between those years. Samples were therefore pooled in 4 groups of years: 1900s, 1950s, 1970s and 2000s (Tables 2 and 3). There was no significant trend within those groups (Pearson’s correlations, all *p*>0.05), except for feather NRP δ^13^C values that decreased in the 1970s (*p* = 0.004). Long-term changes were then investigated by non-parametric comparisons of groups (a Kolmogorov-Smirnov test and multiple comparison tests of mean ranks, the groups being non-equilibrated and variance non homogenous).

In our long-term analyses using historic and contemporary samples, the raw δ^13^C values of penguin feathers were adjusted following [15] to take into account the two effects due to the increase in atmospheric CO_2_ in response to human fossil fuel burning. Firstly, the resulting higher [CO_2_]aq increases in turn phytoplankton fractionation, thus reducing its δ^13^C isotopic values [27]. Secondly, fossil carbon introduced into the atmosphere has a lower δ^13^C than background carbon, thus inducing an accelerating decrease in δ^13^C in the biosphere (the Suess effect, [28]). Importantly, some aspects of our results rely on the appropriate use of these correction factors. Although there are some attempts to quantify and adjust for both these factors, there is considerable uncertainty about the magnitude and spatial variations of both effects [15]. While the model of [CO_2_]_aq_ increase effect is low with a maximum correction factor of 0.16‰ for the period from 1850 to 2007, the Suess effect model might potentially impact the overall temporal trend with a maximum effect of 0.81‰. Suess effect correction factors added to penguin feather δ^13^C values for each species and groups of years were presented in Table S2. The influence of Suess effect correction factors on our long-term analyses depend on the year groups considered. Since these positive correction factors were added to modern values, long-term unadjusted δ^13^C values exhibited a similar and even greater tendency (i.e. decreasing) than adjusted δ^13^C values in the comparison between old (1900s) and more recent values (1970s and 2000s). Conversely, the δ^13^C differences between 1970s and 2000s samples might be exacerbated by the addition of Suess correction factors. We cannot rule out that the true effects are precisely evaluated, but there are increasing evidences of a strong effect of human fossil fuel burning on marine carbon isotopic ratios [29], [30], [31]. No correction factor was used in the short-term analyses, because the calculated δ^13^C change between 2006 and 2007 due to both Suess and aqueous CO_2_ effects was negligible (∼0.01‰).

## Results

### Short-term isotopic variations

All penguin species, but one (SRP) showed distinct inter-annual moulting isotopic niches (Table 1). Depending on species, δ^13^C values were 0.1–1.3‰ higher in 2007 than in 2006. Surface Chl a concentrations were also 5–19% higher in 2007 than in 2006 in the different water masses of the southern Indian Ocean (from high-Antarctica to the subtropics), as indicated by the positive differences in Chl a concentration between 2007 and 2006 (Fig. 2). Interestingly, individual inter-annual differences in penguin feather δ^13^C values were positively correlated with inter-annual differences in surface Chl a concentrations (Fig. 2, Pearson’s correlation, *r* = 0.354 and *p* = 0.003).

Feather δ^15^N values of three penguin species (AP, GP and NRP) were significantly ∼1‰ higher in 2007 than in 2006, while the three remaining species (KP, MP and SRP) did not show any significant inter-annual differences in their feather nitrogen signatures (Table 1).

### Long-term isotopic variations

Corrected feather δ^13^C values of subtropical (NRP) and three out of four subantarctic species (GP, MP, SRP) had lower feather δ^13^C values in the 1970s than in the 2000s (Table 2; Kolmogorov-Smirnov tests, both *p*<0.005; multiple comparison tests of mean ranks, *p* = 0.004 and 0.001, respectively). Older historic NRP samples (1900s) showed higher δ^13^C values than in 1970s (multiple comparison tests of mean ranks, *p* = 0.003) and no significant difference with contemporary (2000s) samples (multiple comparison tests of mean ranks, *p* = 1.000). For SRP, only two samples were available in 1900s but they confirmed latest analyses. In contrast, the two Antarctic species (EP and AP) and KP did not show significant temporal change between 1950s and 2000s (EP and AP: multiple comparison tests of mean ranks, *p*>0.5; KP: Kolmogorov-Smirnov test, *p* = 0.100).

**Figure 3:**
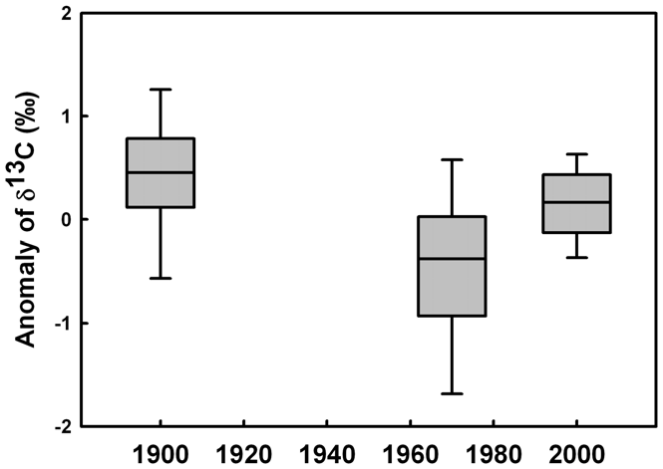
Long-term trend in anomalies of feather δ^13^C values (corrected by Suess and phytoplankton fractionation effects). Anomalies were calculated independently for each subantarctic and subtropical penguin species (except the king penguin, see text for details). In the 1970s, δ^13^C anomalies were significantly lower than in the 1900s and 2000s (p<0.0001 and p<0.001, respectively).

In order to perform a global analysis including all subantarctic (except KP) and subtropical species, we calculated δ^13^C anomalies from δ^13^C values, i.e. the mean feather δ^13^C value was subtracted from each δ^13^C value for a given penguin species. Latitudinal effect on δ^13^C value [32], [33] was consequently blurred and all species can be compared in a single analysis. Anomalies were significantly lower in the 1970s (−0.5±0.5‰) than in the 1900s (0.4±0.8‰) and 2000s (0.1±0.4‰) (multiple comparison tests of mean ranks, *p*<0.0001 and *p*<0.001, respectively) (Fig. 3).

Historic feather δ^15^N values were significant higher than contemporary samples for EP (in the 1950s and 1970s) and MP (in the 1970s) penguins (EP: multiple comparison tests of mean ranks, *p* = 0.049, 0.016 and 0.042; MP: Kolmogorov-Smirnov test, *p*<0.025). The five other penguin species did not present significant change in their feather δ^15^N values over time (statistics not shown; Table 3).

## Discussion

In a first methodological step, we investigated short-term (contemporary) isotopic variations of penguin feathers and showed for the first time concurrent variations in consumer δ^13^C values and surface Chl a concentration, a proxy of marine primary productivity. In a second exploratory step, historic changes in penguin isotopic niche were investigated and the relationship relating penguin δ^13^C values to ecosystem productivity used to depict long-term trends in the pelagic ecosystem of the southern Indian Ocean.

### Short-term isotopic variations

Little is known about the penguin biology during the pre-moult period, because birds spend 2–3 weeks away at sea to build up energy reserves, hence, the use of indirect methods to investigate their feeding ecology at this time [34], [35], [36]. Overall, the isotopic niches of moulting penguins agree well with previous blood isotopic investigations and birds’ foraging areas and diets during the breeding period. Species differences in feather δ^13^C values reflect the oceanic δ^13^C latitudinal gradient at the base of the food web. The low δ^13^C values of EP and AP, intermediate values of KP, MP and SRP, and high value of NRP are representative of Antarctic, subantarctic and subtropical waters, respectively [32], [33]. Otherwise, the high carbon signature of GP is related to its coastal habits, and thus illustrates the inshore-offshore gradient in organism δ^13^C values [33]. Species differences in feather δ^15^N values reflect mainly their dietary habits, with EP, KP and GP preying primarily upon fish, and thus higher in the food web than AP, MP and SRP that feed on both fish and crustaceans [20], [21]. Finally, the high nitrogen signature of NRP results from the higher δ^15^N value characterizing the baseline level in warm subtropical waters [32], [33].

Carbon and nitrogen isotopic signatures of penguin feathers presented substantial inter-annual variations. Such short-term variations were rarely investigated and they were generally interpreted in terms of changes in the birds’ feeding ecology [35]. We think that this explanation (*i.e.* changes in foraging locations) can be rejected for penguin δ^13^C values for two reasons. Firstly, penguins are spatio-temporally constrained in the vicinity of their colonies during the pre-moulting foraging period, because they have to moult ashore at their breeding sites afterwards. Secondly, the same trend in feather δ^13^C values was found in 5 penguin species (that span a large geographical range), thus strongly suggesting a broad-scale explanation rather than species-specific changes in foraging habitats. Indeed, surface Chl a concentration was higher in 2007 than in 2006 over a broad latitudinal gradient that includes high-Antarctic, subantarctic and subtropical waters, therefore indicating that the carrying capacity of the southern Indian Ocean was higher in 2007 than in 2006. A major finding of the present work is that the inter-annual differences in penguin feather δ^13^C values and surface Chl a concentrations were positively correlated.

Phytoplankton carbon fractionation is an inverse function of cell growth rate. Hence, as phytoplankton grows more rapidly, its δ^13^C values increase [37]. Since any change affecting the carbon isotopic baseline level is subsequently carried out throughout the food web [32], [33], δ^13^C values of top predators have been used as bio-indicators of ecosystem productivity [18]. Our data reinforce this approach by suggesting a causal link between δ^13^C values of top predators and productivity of the pelagic ecosystem. Our analysis was performed on a significant number of penguin species, but during a relatively short temporal window (two consecutive years only). Clearly, the relationship between penguin δ^13^C value and surface Chl a concentration needs to be investigated during a longer period. Also, the higher inter-annual change of king penguin δ^13^C values (Fig. 2) suggests possible inter-species variations in the penguins’ responses to change in marine productivity that also call for further investigations. Hopefully, feathers can be easily collected year after year in the field on different species with a minimum disturbance of the birds. Hence, δ^13^C values of penguin feathers have therefore the potential to be used to monitor the carrying capacity of the Southern Ocean over the long term.

### Long-term isotopic variations

The southern Indian Ocean was marked by a temperature regime shift in the 1970s associated with population changes of predators, including penguins [4]. A change in atmospheric circulation during the 1970s brought warmth and moisture until Antarctic coast [7] that probably explain the air temperatures increase between the mid 1960s and mid 1980s at various localities, including Adélie Land, Crozet and Amsterdam Islands [4], [6]. In the present work, penguin historic collection was not continuous over the last decades, but feather isotopic data illustrate key time-periods, with sampling in the 1970s reflecting the dynamic period of temperature decrease, while older (1900s and 1950s) and contemporary (2000s) samples correspond to the pre- and post- regime shift periods, respectively. Overall, isotopic niches of penguins did not change drastically over time, and segregating mechanisms operating in historic times were the same than those operating today [20], [21]. Some consistent isotopic changes were however depicted and they can be related either to species (δ^15^N) or broad geographical areas (δ^13^C).

Two penguin species (EP and MP) showed long-term temporal changes in their feather δ^15^N values. Feather δ^15^N changes were species-specific, suggesting temporal variations in penguin diet rather than in ecosystem baseline δ^15^N levels. Short-term δ^15^N changes were previously observed in AP. They were interpreted as varying proportions of fish and krill in their diet, with the higher δ^15^N value of fish over krill inducing a corresponding increase in predator δ^15^N signature [21], [35]. Our data therefore suggest that EP and MP fed less on fish and more on crustaceans in 2000s than in historic times.

A main finding of the present work is that feather δ^13^C values of Antarctic penguins did not change over time, while the carbon isotopic signature of subantarctic and subtropical species presented lower values in the 1970s during the temperature regime shift. Similarly to short-term variations, long-term variations in δ^13^C values can be explained in two ways, a change in penguin foraging location and/or in primary productivity of the environment. The consistent δ^13^C changes over species and localities strongly support the latter hypothesis. The ecosystemic explanation includes no major changes in the carrying capacity of high-Antarctic waters in the vicinity of Adélie Land. By contrast, historic and contemporary penguin δ^13^C values suggest a lower carrying capacity of the pelagic ecosystem in both subantarctic (Crozet) and subtropical (Amsterdam) waters in the 1970s when compared to older and present times. Such a trend was also observed from feather δ^13^C values of SRP from Kerguelen Islands, i.e. another subantarctic locality from the southern Indian Ocean (data from [15]). Unfortunately, no information is available on changes in primary productivity of the southern Indian Ocean over the last decades, thus precluding any comparison with previous oceanographic investigations.

Penguin δ^13^C values therefore suggest that the decrease in carrying capacity of the southern Indian Ocean could be the underlying cause linking the temperature regime shift and the predator population declines in the Subtropical and Subantarctic zones. Within that context, how to explain the population decreases of Antarctic penguins in Adélie Land, while their δ^13^C values suggest no major change in primary productivity? In Adélie Land, warming in the 1970s was associated with a regional transient decrease in sea ice extent [38], which is known to negatively impact the population of Antarctic krill, a keystone organism in the pelagic food web [39]. Since AP feeds mainly on krill and EP on krill-eating prey [21], lower krill biomass is the likely explanation of the declines in penguin populations in Adélie land. Overall, the study therefore suggests that a lower secondary productivity, and thus a decrease in food availability, was the main driving force explaining penguin population declines in various localities of the southern Indian Ocean during the regime shift. In contrast to other penguins, KP increased its population at Crozet (and elsewhere) at this time, probably a long-term response from the cessation of past exploitation during the nineteenth century [40]. Feather δ^13^C and δ^15^N values of KP did not show temporal variations, suggesting neither species-specific nor ecosystemic change during the study period for this highly specialized penguin that forages on myctophid fish at the Polar Front [41], [42].

The decrease in ecosystem productivity in subantarctic and subtropical zones infer from penguin isotopic data was probably also involved in population changes of pinnipeds, albatrosses and petrels that occurred during the regime shift [4]. However, more work is needed before extrapolating an ecosystemic explanation to Procellariiformes, because firstly they are very wide-ranging organisms when compared to penguins, and secondly their populations are not only impacted by climatic changes, but also by anthropogenic factors, such as positive and negative effects due to fisheries [43]. The present study illustrates the usefulness of the isotopic tool to help disentangling among multi-factors affecting predator populations. It highlights the need of investigating several species spanning broad geographic areas and the utility of developing long-term tissue sampling and data bases on isotopic signature of key marine organisms.

## Supporting Information

Table S1 **Details of inter-annual sea surface chlorophyll a calculation for each penguin species.**
(DOC)

Table S2 **Suess effect correction factors (‰) added to feather δ^13^C values.** They are presented for each penguin species and each group of sampling years.
(DOC)

## References

[1] Walther GR, Post E, Convey P, Menzel A, Parmesan C (2002). Ecological responses to recent climate change.. Nature.

[2] Baillie JEM, Hilton-Taylor C, Stuart SN (2004). 2004 IUCN Red List of Threatened Species. A Global Species Assessment..

[3] Croxall JP, Prince PA, Rothery P, Wood AG, Robertson G, Gales R (1998). Population changes in albatrosses at South Georgia.. Albatross biology and conservation.

[4] Weimerskirch H, Inchausti P, Guinet C, Barbraud C (2003). Trends in bird and seal populations as indicators of a system shift in the Southern Ocean.. Antarct Sci.

[5] Delord K, Besson D, Barbraud C, Weimerskirch H (2008). Population trends in a community of large Procellariiforms of Indian Ocean: potential effects of environment and fisheries interactions.. Biol Conserv.

[6] Jenouvrier S, Weimerskirch H, Barbraud C, Park YH, Cazelles B (2005). Evidence of a shift in cyclicity of Antarctic seabirds dynamics link to climate.. Proc R Soc B.

[7] Masson-Delmotte V, Delmotte V, Morgan V, Etheridge D, Van Ommen T (2003). Recent southern Indian Ocean climate variability inferred from a Law Dome ice core, new insights for the interpretation of coastal Antarctic isotopic records.. Clim Dyn.

[8] Croxall JP, Trathan PN, Murphy EJ (2002). Environmental change and Antarctic seabirds populations.. Science.

[9] Thompson DR, Furness RW, Lewis SA (1995). Diets and long-term changes in δ^15^N and δ^13^C values in northern fulmars *Fulmarus glacialis* from two northeast Atlantic colonies.. Mar Ecol Prog Ser.

[10] Hirons AC, Schell DM, Finney BP (2001). Temporal records of δ^13^C and δ^15^N in North Pacific pinnipeds: inferences regarding environmental change and diet.. Oecologia.

[11] Hobson KA, Sinclair EH, York AE, Thomason JR, Merrick RE (2004). Retrospective isotopic analyses of Steller sea lion tooth annuli and seabird feathers: a cross-taxa approach to investigating regime and dietary shifts in the Gulf of Alaska.. Mar Mammal Sci.

[12] Emslie SD, Patterson WP (2007). Abrupt recent shift in δ^13^C and δ^15^N values in Adélie penguin eggshell in Antarctica.. Proc Nati Acad Sci U S A.

[13] Chamberlain CP, Waldbauer JR, Fox-Dobbs K, Newsome SD, Koch PL (2005). Pleistocene to recent dietary shifts in California Condors.. Proc Nati Acad Sci U S A.

[14] Becker BH, Beissinger SR (2006). Centennial decline in the trophic level of an endangered seabird after fisheries decline.. Conserv Bio.

[15] Hilton GM, Thompson DR, Sagar PM, Cuthbert RJ, Cherel Y (2006). A stable isotopic investigation into the causes of decline in a sub-Antarctic predator, the rockhopper penguin *Eudyptes chrysocome*.. Global Change Biol.

[16] Rubenstein DR, Hobson KA (2004). From birds to butterflies: animal movement patterns and stable isotopes.. Trends Ecol Evol.

[17] Newsome SD, Martinez del Rio C, Bearhop S, Phillips DL (2007). A niche for isotopic ecology.. Front Ecol Environ.

[18] Schell DM (2000). Declining carrying capacity in the Bering Sea: isotopic evidence from whale baleen.. Limnol Oceanogr.

[19] Cherel Y, Charrassin JB, Challet E (1994). Energy and protein requirements for molt in the king penguin *Aptenodytes patagonicus*.. Am J Physiol.

[20] Cherel Y, Hobson KA, Guinet C, Vanpé C (2007). Stable isotopes document seasonal changes in trophic niches and winter foraging individual specialisation in diving predators from the Southern Ocean.. J Anim Ecol.

[21] Cherel Y (2008). Isotopic niches of emperor and Adélie penguins in Adélie Land, Antarctica.. Mar Biol.

[22] Moore JK, Abbott MR (2000). Phytoplankton chlorophyll distributions and primary production in the Southern Ocean.. J Geophys Res.

[23] Behrenfeld MJ, O’Malley RT, Siegel DA, McClain CR, Sarmiento JL (2006). Climate-driven trends in contemporary ocean productivity.. Nature.

[24] Park YH, Gambéroni L (1997). Cross-frontal exchange of Antarctic Intermediate Water and Antarctic Bottom Water in the Crozet Basin.. Deep-Sea Res II.

[25] Michalik A, McGill RAR, Furness RW, Eggers T, van Noordwijk HJ (2010). Black and white – does melanin change the bulk carbon and nitrogen isotope values of feathers?. Rapid Commun Mass Spectrom.

[26] Cherel Y, Hobson KA, Bailleul F, Groscolas R (2005). Nutrition, physiology, and stable isotopes: new information from fasting and molting penguins.. Ecology.

[27] Rau GH, Takahashi T, Marais DJD, Repeta DJ, Martin JH (1992). The relationship between organic matter δ^13^C and [CO2_(aq)_] in ocean surface water: data from a JGOFS site in northeast Atlantic Ocean and a model.. Geochim Cosmochim Acta.

[28] Keeling CD (1979). The Suess effect: ^13^Carbon and ^14^Carbon interactions.. Environm Intern.

[29] Gruber N, Keeling CD, Bacastow RB, Guenther PR, Lueker TJ (1999). Spatiotemporal patterns of carbon-13 in the global surface oceans and the oceanic Suess Effect.. Global Biogeochem Cycles.

[30] Sonnerup RE, Quay PD, McNichol AP (2000). The Indian Ocean ^13^C Suess Effect.. Global Biogeochem Cycles,.

[31] McNeil BI, Matear RJ, Tilbrook B (2001). Does carbon 13 track anthropogenic CO_2_ in the Southern Ocean?. Global Biogeochem Cycles.

[32] Jaeger A, Lecomte VJ, Weimerskirch H, Richard P, Cherel Y (2010). Seabird satellite tracking validates the use of latitudinal isoscapes to depict predators' foraging areas in the Southern Ocean.. Rapid Commun Mass Spectrom.

[33] Cherel Y, Hobson KA (2007). Geographical variation in carbon stable isotope signatures of marine predators: a tool to investigate their foraging areas in the Southern Ocean.. Mar Ecol Prog Ser.

[34] Raclot T, Groscolas R, Cherel Y (1998). Fatty acid evidence for the importance of myctophid fishes in the diet of king penguins, *Aptenodytes patagonicus*.. Mar Biol.

[35] Tierney M, Southwell C, Emmerson LM, Hindell MA (2008). Evaluating and using stable-isotope analysis to infer diet composition and foraging ecology of Adélie penguins *Pygoscelis adeliae*.. Mar Ecol Prog Ser.

[36] Cherel Y, Fontaine C, Richard P, Labat JP (2010). Isotopic niches and trophic levels of myctophid fishes and their predators in the Southern Ocean.. Limnol Oceanogr.

[37] Laws EA, Popp BN, Bidigare RR, Kennicutt MC, Macko SA (1995). Dependence of phytoplankton carbon isotopic composition on growth rate and [CO2]aq: theoretical considerations and experimental results.. Geochim Cosmochim Acta.

[38] Barbraud C, Weimerskirch H (2001). Emperor penguins and climate change.. Nature.

[39] Nicol S (2006). Krill, currents, and sea-ice: *Euphausia superba* and its changing environment.. BioSci.

[40] Delord K, Barbraud C, Weimerskirch H (2004). Long-term trends in the population size of king penguins at Crozet archipelago: environmental variability and density dependence?. Polar Biol.

[41] Cherel Y, Verdon C, Ridoux V (1993). Seasonal importance of oceanic myctophids in king penguin diet at Crozet Islands.. Polar Biol.

[42] Charrassin J-B, Bost CA (2001). Utilisation of the oceanic habitat by king penguins over the annual cycle. Mar Ecol Prog Ser.

[43] Rolland V, Barbraud C, Weimerskirch H (2008). Combined effects of fisheries and climate on a migratory long-lived marine predator.. J Appl Ecol.

