# Supplementary material for: Isotopic Investigation of Contemporary and Historic Changes in Penguin Trophic Niches and Carrying Capacity of the Southern Indian Ocean
Source: PLoS One. 2011 Feb 2;6(2):e16484. doi: 10.1371/journal.pone.0016484 (PMC3032774; doi:10.1371/journal.pone.0016484)
Supplement: Table S1 — Details of inter-annual sea surface chlorophyll a calculation for each penguin species. (DOC) [file pone.0016484.s001.doc]

**Table S1. Details of inter-annual sea surface chlorophyll a calculation for each penguin species.**

| **Species** | **Location** | **Water mass zone** | **Latitudinal range** | **Longitudinal range** | **Month range** | **Chla_2007_ - Chla_2006_ (mg.m^-3^)** |
| --- | --- | --- | --- | --- | --- | --- |
| AP | Adélie Land | Antarctic | 50° - 70° S | 130° - 150° E | Jan – Feb | 0.035 |
| KP | Crozet | Subantarctic | 40° - 60° S | 40° - 60° E | Aug – Sept | 0.020 |
| GP | Crozet | Subantarctic | 40° - 60° S | 40° - 60° E | Dec – Jan | 0.018 |
| MP | Crozet | Subantarctic | 40° - 60° S | 40° - 60° E | Mar – Apr | 0.006 |
| SRP | Crozet | Subantarctic | 40° - 60° S | 40° - 60° E | Mar – Apr | 0.006 |
| NRP | Amsterdam | Subtropical | 30° - 50° S | 70° - 0° E | Feb -Mar | 0.021 |
