# Supplementary material for: Isotopic Investigation of Contemporary and Historic Changes in Penguin Trophic Niches and Carrying Capacity of the Southern Indian Ocean
Source: PLoS One. 2011 Feb 2;6(2):e16484. doi: 10.1371/journal.pone.0016484 (PMC3032774; doi:10.1371/journal.pone.0016484)
Supplement: Table S2 — Suess effect correction factors (‰) added to feather δ13C values. They are presented for each penguin species and each group of sampling years. (DOC) [file pone.0016484.s002.doc]

**Table S2. Suess effect correction factors (‰) added to feather δ^13^C values.** They are presented for each penguin species and each group of sampling years.

| **Species** | **1900s** | **1950s** | **1970s** | **2000s** |
| --- | --- | --- | --- | --- |
| EP | 0.00 | 0.02 | 0.04 | 0.09 |
| AP | 0.00 | 0.02 | 0.04 | 0.09 |
| KP | 0.03 | 0.16 | 0.28 | 0.62 |
| GP | 0.03 | 0.16 | 0.28 | 0.62 |
| MP | 0.03 | 0.16 | 0.28 | 0.62 |
| SRP | 0.03 | 0.16 | 0.28 | 0.62 |
| NRP | 0.03 | 0.18 | 0.32 | 0.72 |
